# Associations of greenness, greyness and air pollution exposure with children’s health: a cross-sectional study in Southern Italy

**DOI:** 10.1186/s12940-018-0430-x

**Published:** 2018-12-05

**Authors:** Giovanna Cilluffo, Giuliana Ferrante, Salvatore Fasola, Laura Montalbano, Velia Malizia, Alessandro Piscini, Vito Romaniello, Malvina Silvestri, Salvatore Stramondo, Massimo Stafoggia, Andrea Ranzi, Giovanni Viegi, Stefania La Grutta

**Affiliations:** 1 National Research Council, Institute of Biomedicine and Molecular Immunology, via Ugo La Malfa 153, 90146, Palermo, Italy; 2 Department of Economics, Business and Statistical Science, University of Palermo, viale delle Scienze, Ed. 13, 90128 Palermo, Italy; 3 Department of Science for Health Promotion and Mother and Child Care, University of Palermo, via del Vespro 129, 90127 Palermo, Italy; 4 Department of Psychological, Pedagogical and Educational Sciences, University of Palermo, viale delle Scienze, Ed. 15, 90128 Palermo, Italy; 5 National Institute of Geophysics and Volcanology, via di Vigna Murata 605, 00143 Rome, Italy; 6 Department of Epidemiology, Latium Region Health Service, via Cristoforo Colombo, 112, 00147 Rome, Italy; 7 Environmental Health Reference Centre, Regional Agency for Environmental Prevention of Emilia-Romagna, via Braghiroli 63, 41125 Modena, Italy; 8 National Research Council, Institute of Clinical Physiology, via Trieste 41, 56126 Pisa, Italy

**Keywords:** Greenness, Greyness, Asthma, Allergic, Air pollution

## Abstract

**Background:**

Due to the complex interplay among different urban-related exposures, a comprehensive approach is advisable to estimate the health effects. We simultaneously assessed the effect of “green”, “grey” and air pollution exposure on respiratory/allergic conditions and general symptoms in schoolchildren.

**Methods:**

This study involved 219 schoolchildren (8–10 years) of the Municipality of Palermo, Italy. Data were collected through questionnaires self-administered by parents and children. Exposures to greenness and greyness at the home addresses were measured using the normalized difference vegetation index (NDVI), residential surrounding greyness (RSG) and the CORINE land-cover classes (CLC). RSG was defined as the percentage of buffer covered by either industrial, commercial and transport units, or dump and construction sites, or urban fabric related features. Two specific categories of CLC, namely “discontinuous urban fabric - DUF” - and “continuous urban fabric - CUF” - areas were found. Exposure to traffic-related nitrogen dioxide (NO_2_) was assessed using a Land-Use Regression model. A symptom score ranging from 0 to 22 was built by summing affirmative answers to twenty-two questions on symptoms. To avoid multicollinearity, multiple Logistic and Poisson ridge regression models were applied to assess the relationships between environmental factors and self-reported symptoms.

**Results:**

A very low exposure to NDVI ≤0.15 (1st quartile) had a higher odds of nasal symptoms (OR = 1.47, 95% CI [1.07–2.03]). Children living in CUF areas had higher odds of ocular symptoms (OR = 1.49, 95% CI [1.10–2.03]) and general symptoms (OR = 1.18, 95% CI [1.00–1.48]) than children living in DUF areas. Children living in proximity (≤200 m) to High Traffic Roads (HTRs) had increased odds of ocular (OR = 1.68, 95% CI [1.31–2.17]) and nasal symptoms (OR = 1.49, 95% CI [1.12–1.98]). A very high exposure to NO_2_ ≥ 60 μg/m^3^ (4th quartile) was associated with a higher odds of general symptoms (OR = 1.28, 95% CI [1.10–1.48]). No associations were found with RGS. A Poisson ridge regression model on the symptom score showed that children living in proximity to HTRs (≤200 m) had a higher symptoms score (RR = 1.09, 95% CI [1.02–1.17]) than children living > 200 m from HTRs. Children living in CUF areas had a higher symptoms score (RR = 1.11, 95% CI [1.03–1.19]) than children living in DUF areas.

**Conclusions:**

Multiple exposures related to greenness, greyness (measured by CORINE) and air pollution within the urban environment are associated with respiratory/allergic and general symptoms in schoolchildren. No associations were found when considering the individual exposure to greyness measured using the RSG indicator.

**Electronic supplementary material:**

The online version of this article (10.1186/s12940-018-0430-x) contains supplementary material, which is available to authorized users.

## Introduction

In an increasingly urbanized world, more children are living in cities. Indeed, demographic trends indicate that the world’s urban population will double by 2050 [1]. In spite of a number of socioeconomic benefits, urbanization has been associated with adverse health effects mainly due to increasing exposure to air pollution [2]. Children are particularly vulnerable to the impacts of environmental exposures because childhood is a period of rapid growth and development and because children breathe more per body kilogram and are more physically active than adults [3].

Environmental factors play an important role in the worldwide increasing prevalence of respiratory and allergic diseases observed during the last decades [4]. In particular, asthma and rhino-conjunctivitis largely contribute to the global burden of disease, with a global prevalence in schoolchildren ranging from < 5 to > 20% and from 0.8 to 39.7%, respectively [5, 6].

Because of increasing urbanization, there is growing interest in factors affecting environmental exposures within urban settings, such as traffic intensity, household density and natural and green space. All these factors were taken into account. The role of both residential surrounding greenery and proximity to green spaces (i.e. ‘greenery’) on respiratory and allergic symptoms in schoolchildren has so far yielded inconsistent results, likely due to differences in exposure timing or in greenery type among different studies [7–12].

The built environment in urban areas (i.e. ‘grey’ surfaces, which comprise industrial, transport and urban features) appears to have side effects on children’s health, mainly due to increasing exposure to air pollution, noise and high temperatures, lower access to natural environments, and accentuated sedentary life [13–16]. Apart from air pollution, there have been few investigations on the association between “urbanicity” and respiratory and allergic symptoms in childhood [2, 17]. Recently, Tischer et al. in a longitudinal study including 2472 children living in two biogeographic regions (Euro-Siberian and Mediterranean) in Spain reported that a higher amount of residential surrounding greyness increases the risk for bronchitis in the Mediterranean region. In 2018, Tischer et al. did not find statistically significant associations for grey-related exposure in relation to wheezing, asthma and rhinitis in pooled data of four European birth cohorts [18, 19].

Land Use Regression (LUR) models have been used for estimating outdoor air pollution concentrations at the home addresses in order to analyze their association with respiratory health [20]. Significant associations between NO_2_ LUR and non-atopy related asthma and wheezing were found in aged 6–7 years girls and 13–14 years female adolescents from randomly selected schools in Hamilton, Canada [21]. Notably, the association between NO_2_ exposure and health outcomes has been shown not to depend on the type of model used to estimate exposure [22].

A comprehensive estimation of children’s health effects due to the complex interplay among different urban-related exposures is highly advisable. In this connection, only two recent studies have developed a similar approach combining “green” and “grey” indices of exposure [18, 19]. Such studies have never been performed in Italy. Moreover, to date, no cross-sectional studies simultaneously using the three aforementioned characteristics (green, grey and air pollution) have been carried out.

The aim of the present study was to simultaneously evaluate the association between indicators of urban-related environmental exposures, including those for “green”, “grey” and NO_2_ exposure, with respiratory/allergic and general symptoms in schoolchildren from the city of Palermo, Italy.

## Materials and methods

### Study population and design

This study is a part of the “*Giardini per Allergici” (Italian for “Gardens for allergic subjects”)* project carried out by a scientific consortium of three partners: CNR - IBIM Palermo, Municipality of Palermo, and Vivisano Onlus no-profit organization. Palermo is a city of 678,492 inhabitants according to the 2013 registry office, located in the northwest of the island of Sicily, on the Gulf of Palermo in the Tyrrhenian Sea (38°06′56″N 13°21′41″E). It has a Mediterranean climate characterized by hot and dry summers with mild temperatures for the rest of the year. The study site, where the two schools are located, was chosen since it represents one of the two “background” monitoring stations of the city, i.e. considered as the “zero-out” approach for estimating background pollutant concentrations [23].

The study was conducted in a flat suburban area of Palermo (10,89 km^2^) located on the extreme western outskirts of the city (38°07′48″N 13°17′54″E). The suburb lies near the Conca d’Oro basin and it is about 6 km from the sea. This area is characterized by intensive and still ongoing edification, mainly resulting in commercial settlements and public housing, and it is about 2 km from the city dump.

The study involved all schoolchildren (8–10 years of age), attending the 3rd to 5th years of two primary schools in Palermo (“Paolo Borsellino” and “Filippo Raciti”) on April 16th 2013.

The study was approved by the local ethics committee (n°5/2011, *A.O.U.P. “Paolo Giaccone”*, Palermo, Italy), and written informed consent was provided by parents of all participants. Overall, 244 children were eligible; questionnaires were obtained for 219 parents and their children (90% response rate). Of the 219 children taking part in the survey, all had lived since birth at their address.

### Parent and child questionnaires

The SIDRIA (*Studi Italiani sui Disturbi Respiratori nell’Infanzia e l’Ambiente,* the Italian arm of the International Study of Asthma and Allergies in Childhood - ISAAC), self-administered parent questionnaire, regarding sociodemographic characteristics, environmental exposures and personal information about their children was completed by parents at home [24] on April 2013.

A child’s history of “ever wheezing” was defined as a positive answer to the question “Has your child ever had wheezing or whistling in the chest at any time in the past?” A child’s history of “doctor diagnosed asthma” was defined if there was an affirmative response to the question “Has asthma been diagnosed by a doctor?”

Rhino-conjunctivitis was defined as a positive answer to both the questions “Has your child ever had a problem with sneezing, or runny, or blocked nose apart from common colds or flu in the last 12 months?” and “In the past 12 months, has this nose problem been accompanied by itching and/or watering eyes?”

Eczema was defined as positive answers to both the following questions: “In the last 12 months, has your child had an itchy rash which was coming and going for at least 6 months?” and “Has this itchy rash at any time affected any of the following places: folds of elbows, behind the knees, in front of the ankles, under the buttocks, or around the neck, ears, or eyes?”

Current passive smoking exposure was assessed through an affirmative response to the question “Are there smokers at home?” Parental history of allergy was defined as at least one parent with a doctor’s diagnosis of respiratory allergy.

All children completed a modified version of the SIDRIA questionnaire for adolescents by themselves at school, properly supported when necessary. The questionnaire used was mainly focused on assessing the occurrence in the last 4 weeks of twenty-two equally weighted respiratory and/or allergic symptoms - such as ocular (burning, itching, dry, red eyes, swollen eyes, sandy feeling in the eyes), nasal (running, itching, sneezing, blocked nose), pulmonary (dyspnea, breathlessness, wheezing) and general symptoms (dry throat, sore throat, cold feeling, headache, malaise, physical discomfort, excessive fatigue, flu or fever, chills, physical discomfort and fatigue) [25, 26].

### Normalized difference vegetation index (NDVI)

The normalized difference vegetation index (NDVI) is a practical and economical tool to study vegetation cover in order to quantify the amount of vegetation within urban centres [10, 12, 19].

NDVI [27] is based on land surface reflectance, ranging from 0 to 1, where 0 means no vegetation and values close to 1 (0.8–0.9) indicate the highest possible density of green leaves. In particular, the index was derived from visible RED (0.63–0.69 μm) and Near-InfraRed (NIR, 0.76–0.86 μm) bands included in ASTER (*Advanced Spaceborne Thermal Emission and Reflection Radiometer*) multispectral images at 15 m × 15 m spatial resolution.

The NDVI maps were generated using the images acquired on April 16th 2013, starting from the ASTER VNIR surface reflectance level 2 product [28]. ASTER VNIR was atmospherically and topographically corrected according to available climatological data and global digital elevation datasets, respectively [29]. For each house location involved in the survey, the NDVI values are considered. Since the satellite pixel size represents an area of more than 200 square metrs, the greenness indicator associated to a single house is achieved from the pixel containing the child’s home.

### CORINE land-cover classes and residential surrounding greyness

The CORINE (Coordination of information on the environment) framework is a Europe-wide satellite-based inventory of land-cover developed by the European Environmental Agency, in order to create a Geographical Information System (GIS) for providing information on the environment. The CORINE programme categorized land-cover into 44 classes at a scale of 1:100000, updated in 2006. CORINE land-cover classes (CLC) are organized into three hierarchical levels (Level 1: 5 categories; Level 2: 15 categories; Level 3: 44 categories) based on the unit area definition. For each home address, a class was assigned from the 44 categories of Level 3. In our study, three classes were identified. Continuous urban fabric (CUF) is the class in which most of the land is covered by buildings, roads and artificially surfaced areas. Non-linear areas of vegetation and bare soil are exceptional. Discontinuous urban fabric (DUF) is the class in which most of the land is covered by structures like buildings, roads and artificially surfaced areas associated with vegetated areas and bare soil, which occupy discontinuous but significant surfaces. The third class identified is “Coniferous forests with continuous canopy” in which vegetation formation is composed mainly of trees, including shrub and bush understoreys, where coniferous species predominate [30].

To determine residential surrounding greyness, the land-cover nomenclature based on Level 2 within a 300-m buffer around the home address was used. Residential surrounding greyness was classed as the percentage of buffer covered by either industrial, commercial and transport units, or dump and construction sites, or urban fabric related features [19].

### Nitrogen dioxide (NO_2_) concentrations from the LUR model

For each child, exposure to NO_2_ concentration was estimated from a LUR model on the basis of the residential address, by using GIS. The LUR methodology seeks to predict pollution concentration at a given location based on surrounding land characteristics (e.g., land use, traffic intensity, proximity to emission sources, meteorology, etc.). Exposure to traffic related air pollution was assessed for each residential address using an implemented LUR model for NO_2_ and GIS variables of meters of High Traffic Roads (HTRs) (roads with > 10,000 vehicles/day) within 200 m. The European Project ESCAPE (European Study of Cohorts for Air Pollution Effects, www.escapeproject.eu) developed a standardized procedure for LUR implementation that identifies common criteria for selection of sampling sites, definition of GIS predictors, development of multiple regression models [31]. In particular, linear regression models were developed using a supervised stepwise selection procedure, starting from univariate regressions of the corrected annual average concentrations with all available potential predictors following the procedures used previously [32]. The predictor giving the highest adjusted explained variance (adjusted R^2^) was selected for inclusion in the model if the effect direction was consistent with the a priori definition. We then evaluated which remaining predictor variable further improved the adjusted R^2^ in order to select the one achieving the highest gain in adjusted R^2^ and the right effect direction. Subsequent variables were not selected if they changed the effect direction of any previously included variable. This process continued until no more variable with the right effect direction added at least 0.01 (1%) to the adjusted R^2^ of the model. As final step, any variable with a *p*-value above 0.10 was removed from the LUR model. If the Variance Inflation Factor (VIF) was over 3 − indicating collinearity-, the variable with the highest VIF was removed and the model re-evaluated. Cook’s D statistics were used to detect influential observations.

Cook’s D statistics values above 1 were further examined assessing the changes in model coefficients on excluding the responsible site. If removal of this site caused large changes in a specific variable’s coefficient, the modeling procedure was repeated using all sites, without including this variable. Overall, model performance was evaluated by leave-one-out cross validation (LOOCV): each site was sequentially left out from the model while the included variables were left unchanged.

Following ESCAPE study protocol, three 1-week monitoring campaigns were performed in 2010 (winter, summer, intermediate), using 30 passive samplers for NO_2_ measurements. Each passive sampler consists of three Palmes-type tubes where NO_2_ is absorbed with a triethanolamine solution laid on stainless steel meshes [33]. Predictors included in the final model were: traffic-related variables (meters of High Traffic Roads in a 300 m buffer and meters of all streets within 100 m) from local road network data; population density (high density and low density within 5000 and 500 m, respectively); presence of industries in 1 km buffer and of green areas (seminatural) within 300 m, derived from CLC data.

Final LUR model (R^2^ of the model = 0.73; R^2^ of the cross-validation = 0.82) allowed to predict NO_2_ concentrations at the residential address for each children.

Following the ESCAPE study protocol, a monitoring campaign was performed in 2010, using 30 passive samplers for NO_2_ measurements. Each passive sampler consists of three Palmes-type tubes where NO_2_ is absorbed with a triethanolamine solution laid on stainless steel meshes [33].

### Potential confounders or effect modifiers

Gender, age (years), maternal and paternal education, parental history of allergy, breastfeeding, preterm birth, maternal smoking during pregnancy, passive smoke exposure at home, atopy, doctor diagnosed asthma and parental history of allergy were obtained through the questionnaires filled out by parents.

The Family’s Socio-Economic Status (FSES) was computed using both the mother’s and father’s educational levels and occupational status. In detail, FSES was based on a combination of education score [rated on a 7-point rating scale ranging from 1 (less than five years of schooling) to 7 (graduation)] and occupation score [rated on a 9-point scale, ranging from 1 (service workers) to 9 (high-level executives and professionals)]. The sums of the education score multiplied by 5 and of the occupation score multiplied by 3 were calculated. The total score was computed as the average of the scores obtained from both parents and was categorized using the 1st quartile as the threshold [34].

### Statistical analysis

Statistical analyses were performed on data collected from the self-administered parent and child questionnaires. Mean values of quantitative variables were compared between groups using one-way analysis of variance (ANOVA). Differences of categorical variables between the groups were analyzed using Chi-squared tests. Due to multicollinearity problems, logistic ridge multi-exposure regression models [35] were used to assess the relationship between environmental factors and self-reported symptoms.

All models were controlled for gender, age, FSES (categorized as > 49.5, 1st quartile), atopy, doctor diagnosed asthma, parental history of allergy and preterm birth. Confounders were selected starting from the model with environmental factors and adding each confounder term one at a time.

In general, the ridge regression method is the most widely applied solution for addressing problems of multicollinearity [36], also showing better performance than other approaches when the sample size is small [37]. It implies adding a small positive constant (λ), i.e. the ridge parameter, to the main diagonal elements of the information matrix. The ridge parameter was selected using likelihood cross-validation [38].

The sum of affirmative answers to twenty-two equally weighted questions on symptoms was used to build a symptom score ranging from 0 to 22, as already shown in other studies [39, 40]. The final score was used as the response variable in a Poisson multi-exposure model with ridge penalization. Associations were expressed in terms of estimated rate ratios (RR) and 95% Confidence Intervals (CIs). The highest exposure to NDVI (> 0.15401, 1st quartile) and the lowest exposure to NO_2_ (< 60.21, 4th quartile) were used as the reference category. The variable RSG was dichotomized as follows: “yes”, if the individual buffer was covered 100% by grey, “no” otherwise.

Given the number of subjects (*n* = 219), the study had 84% power to detect a small standardized effect size of 0.2. This calculation was based on a two-sided proportion test with a type I error level of 5%.

Analyses were performed using R 3.1.0 software. A *p*-value< 0.05 was considered statistically significant.

## Results

Parents’ questionnaires on children’s characteristics (no. = 219) were used to obtain the data presented in Table 1. Parental history of allergy was found in 35.56%; wheezing sometimes was found in 25.11%; childhood history of doctor-diagnosed asthma was only found in 5.48%; rhino-conjunctivitis was reported in 26.94% and eczema in 8.68%. The median (IQR) exposures were 0.233 (0.20) and 49.98 (28.21) for NDVI and NO_2_, respectively. 54.33% of children lived ≤200 m from HTRs.

**Table 1:** Characteristics of study population from parent questionnaires, according to host and environmental factors

	All
*n*	219
Personal factors	
Female, *n* (%)	135 (61.6%)
Age, mean (SD), years	8.98 (0.9)
Weight, mean (SD), kilograms	34.51 (7.61)
Height, mean (SD), centimeters	134.92 (7.77)
Parental history of allergy, *n* (%)	78 (35.56%)
Maternal smoke in pregnancy, *n* (%)	26 (11.87%)
Preterm born, *n* (%)	69 (31.50%)
Wheezing ever, *n* (%)	55 (25.11%)
Doctor diagnosed asthma, *n* (%)	12 (5.48%)
Rhino-conjunctivitis, *n* (%)	59 (26.94%)
Eczema, *n* (%)	19 (8.68%)
Atopy, *n* (%)	12 (5.48%)
Environmental factors	
NDVI (15-m buffer)	
Median (IQR)	0.233 (0.20)
CLC levels, *n* (%)	
dicontinuous urban fabric (DUF)	184 (84.02%)
continuous urban fabric (CUF)	34 (15.53%)
coniferous forests	1 (0.05%)
Residential surrounding greyness (300-m buffer), *n* (%)	134 (61.19%)
HTRs ≤200 m, *n* (%)	119 (54.33%)
NO_2_	
Median (IQR), μg/m^3^	49.98 (28.21)
Current passive smoke exposure, *n* (%)	175 (79.91%)
FSES	
Median (IQR)	52 (5)

Data are expressed as n (%) or mean (SD), *NDVI* normalized difference vegetation index, *CLC* CORINE land-cover, *HTRs* High Traffic Roads, *NO*_*2*_ Nitrogen Dioxide, *FSES* Family’s Socio-Economic Status

Information collected from children questionnaires (*n* = 219) was used to obtain data on self-reported symptoms in the last 4 weeks (Table 2).

**Table 2:** Self-reported symptoms in the last 4 weeks

	All
*n*	219
Ocular symptoms, *n* (%)	130 (59.36%)
burned eyes	82 (37.44%)
itching eyes	71 (32.42%)
dry eyes	22 (10.05%)
sensation of sandy feeling in the eyes	74 (33.79%)
red eyes	64 (29.22%)
swollen eyes	31 (14.16%)
Nasal symptoms, *n* (%)	167 (76.26%)
running nose	89 (40.64%)
itching nose	82 (37.44%)
sneezing	141 (64.38%)
blocked nose	106 (48.4%)
Pulmonary symptoms, *n* (%)	48 (21.92%)
dyspnea	38 (17.35%)
breathlessness	16 (7.31%)
wheezing	19 (8.68%)
General symptoms, *n* (%)	200 (91.32%)
dry throat	89 (40.64%)
sore throat	112 (51.14%)
cold feeling	84 (38.36%)
headache	159 (72.60%)
malaise	68 (31.05%)
physical discomfort	116 (52.97%)
excessive fatigue	132 (60.27%)
flu or fever	93 (42.47%)
chills of cold	128 (58.44%)
Symptom score	
0 (no symptoms)	7 (3.20%)
1–3 symptoms	30 (13.70%)
4–6 symptoms	51 (23.30%)
≥ 7 symptoms	131 (59.80%)

**Fig. 1:**
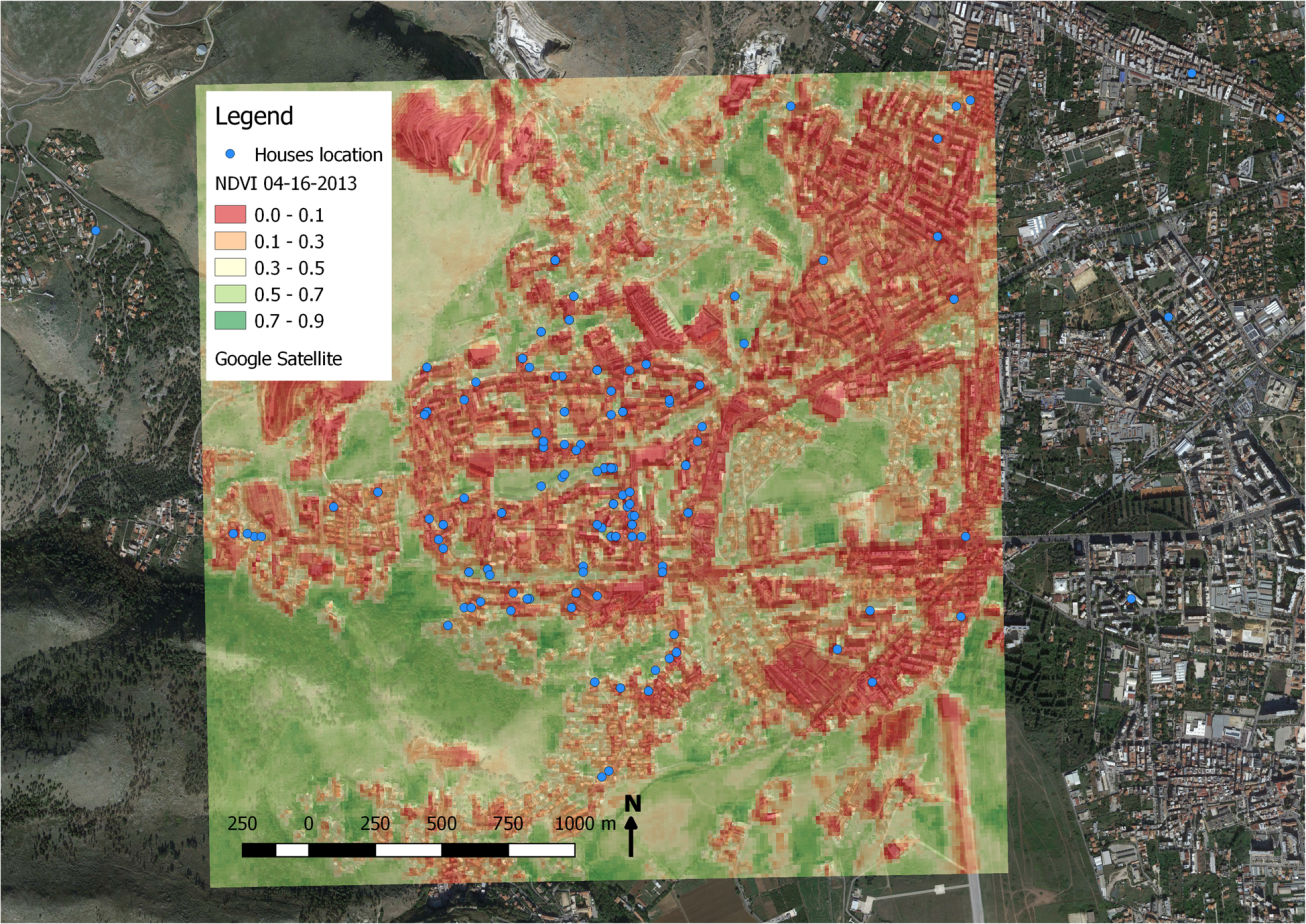
NDVI index of geo-coded children for April 16, 2013, obtained from ASTER optical images. Values greater than 0.5 indicate healthy vegetation. Blue points indicate the residence of each child; colors from red (low exposure) to green (high exposure) indicate the intensity of NDVI

**Fig. 2:**
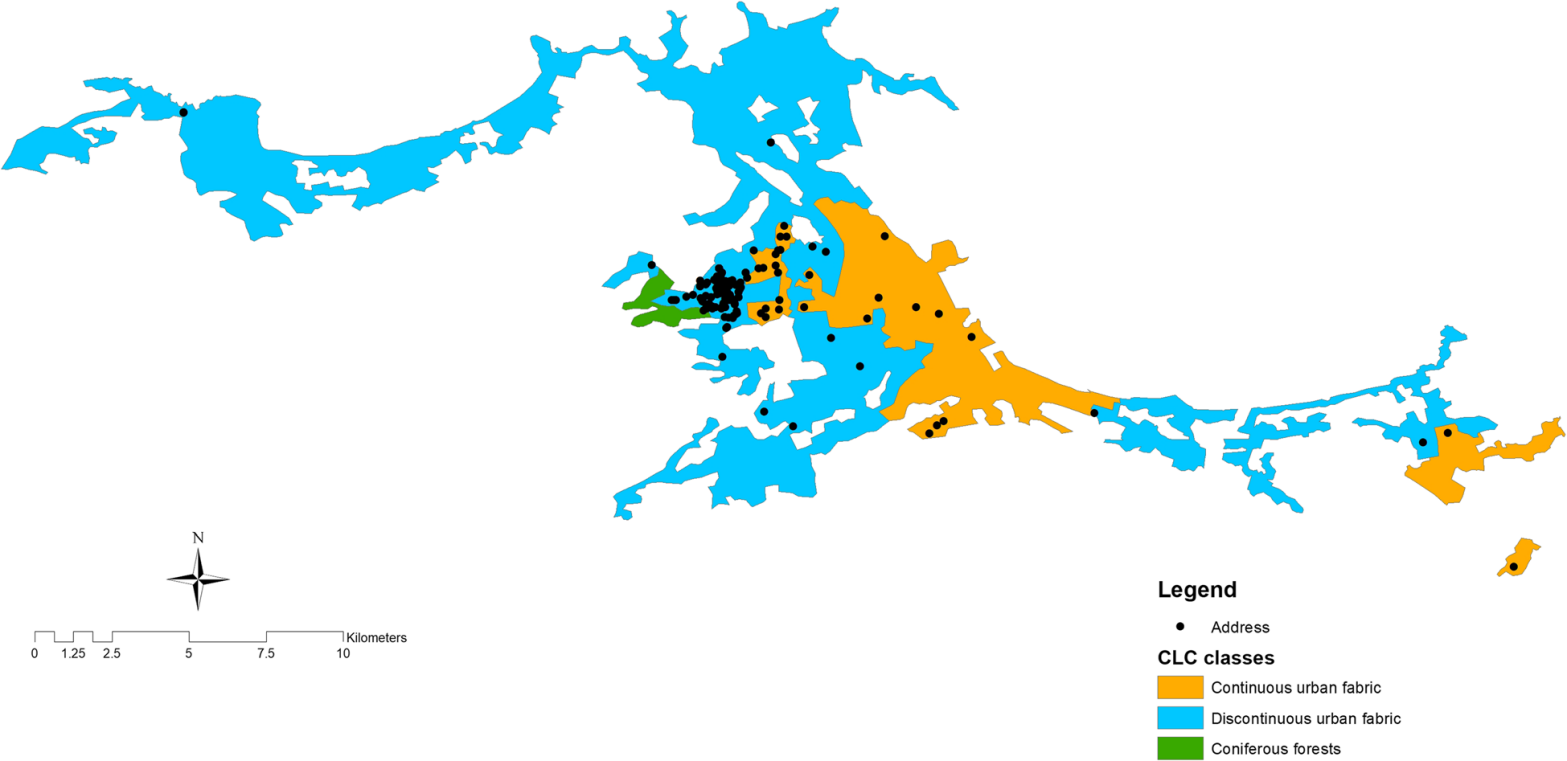
CLC category map of geo-coded children. Black points indicate the residence of each child, and colored polygons identify the CORINE land cover categories

Figure 1 illustrates the NDVI distribution of geo-coded children living at their home addresses. Each dot on the map represents a subject’s house. The map indicates the NDVI values generated using the images acquired on April 16th 2013. Children lived very close to each other and had similar exposure levels. Indeed, most children had an NDVI< 0.3 (*n* = 155) whereas few children had an NDVI> 0.50 (*n* = 26). Figure 2 depicts the land cover map of the area derived by the CORINE database. The map was drawn using the Esri ArcMap 9.3 software. Children’s houses fell into two specific categories, namely “discontinuous urban fabric, DUF” and “continuous urban fabric, CUF” areas; only one house was excluded from the analysis since it fell into a coniferous forest with continuous canopy not on mire. Black points indicate the residence of each child, while colored polygons identify the CORINE land cover categories, as shown in the legend of the Fig.

A significant positive correlation was found between NO_2_ and greyness (*ρ* = 0.54, *p* < 0.001) and a negative one at a borderline level between NO_2_ and NDVI (*ρ* = − 0.13, *p* = 0.05). A higher level of NO_2_ was found in CUF (55.91 vs. 44.01 *p* = 0.001) compared to DUF, whereas a higher level of NDVI was recorded in DUF (0.28 vs. 0.21, *p* = 0.02) than in CUF. No associations were found between greyness and CLC and NDVI.

Estimated odds ratios from multivariable Logistic ridge regression models for the odds of ocular, pulmonary, nasal and general symptoms are reported in Table 3. Children with a very low exposure to NDVI ≤0.15 (1st quartile) had a higher odds of nasal symptoms (OR = 1.47, 95%CI [1.07–2.03]). Children living in CUF areas had higher odds of ocular symptoms (OR = 1.49, 95% CI [1.10–2.03]) and general symptoms (OR = 1.18, 95% CI [1.00–1.48]) than children living in DUF areas. Children living in proximity (≤200 m) to HTRs had increased odds of ocular (OR = 1.68, 95% CI [1.31–2.17]) and nasal (OR = 1.49, 95% CI [1.12–1.98]) symptoms. Very high exposure to NO_2_ ≥ 60 μg/m^3^ (4th quartile) was associated with higher odds of general symptoms (OR = 1.28, 95% CI [1.10–1.48]). No significant associations were found between the exposures and the odds of pulmonary symptoms. No associations were found between all considered symptoms and RGS.

**Table 3:** Risk factors for self-reported ocular, nasal, pulmonary and general symptoms from children questionnaires: estimated odds ratios (OR) and 95% confidence intervals (95% CI) from multivariable logistic ridge regression models

	Ocular symptoms	Nasal symptoms	Pulmonary symptoms	General symptoms
	Adjusted* OR [95% CI]	Adjusted* OR [95% CI]	Adjusted* OR [95% CI]	Adjusted* OR [95% CI]
NDVI > 0.15 (reference)	1.00	1.00	1.00	1.00
NDVI ≤0.15 (1st quartile)	1.17 [0.88–1.54]	**1.47 [1.07–2.03]**	0.98 [0.79–1.21]	0.99 [0.80–1.23]
DUF (reference)	1.00	1.00	1.00	1.00
CUF	**1.49 [1.10–2.03]**	1.11 [0.73–1.70]	0.97 [0.75–1.25]	**1.18 [1.00–1.48]**
No RSG (reference)	1.00	1.00	1.00	1.00
RSG (300-m buffer)	0.98 [0.76–1.26]	1.16 [0.86–1.55]	0.98 [0.79–1.21]	1.03 [0.84–1.28]
HTRs > 200 m (reference)	1.00	1.00	1.00	1.00
HTRs≤200 m	**1.68 [1.31–2.17]**	**1.49 [1.12–1.98]**	0.91 [0.75–1.10]	1.12 [0.92–1.36]
NO_2_ < 60 μg/m^3^ (reference)	1.00	1.00	1.00	1.00
NO_2_ ≥ 60 μg/m^3^ (4th quartile)	1.13 [0.85–1.51]	1.12 [0.81–1.55]	1.04 [0.84–1.29]	**1.28 [1.10–1.48]**

Definition of abbreviations: *NDVI* Normalized Difference Vegetation Index, *DUF* Discontinuous Urban Fabric, *CUF* Continuous Urban Fabric, *RSG* Residential Surrounding Greyness, *HTRs* High traffic roads, *NO*_*2*_ Nitrogen dioxide, *FSES* Family’s Socio-Economic Status. Significant effects are in bold. Values represent odds ratios, with 95% confidence intervals shown in parentheses. *Accounting for gender, age, FSES, atopy, doctor diagnosed asthma, parental history of allergy and preterm born. Reference group: Female, NDVI > 0.15; Discontinuous Urban Fabric, No RSG, HTR > 200 m, NO_2_ < 60 μg, FSES > 49, no sensitization, no asthma, no parental history of allergy and term born

Unadjusted analyses are reported in the Additional file 1: Table S1.

Logistic ridge regression models performed using the full quartile for NDVI and NO_2_ are shown in Additional file 1: Table S2.

**Fig. 3:**
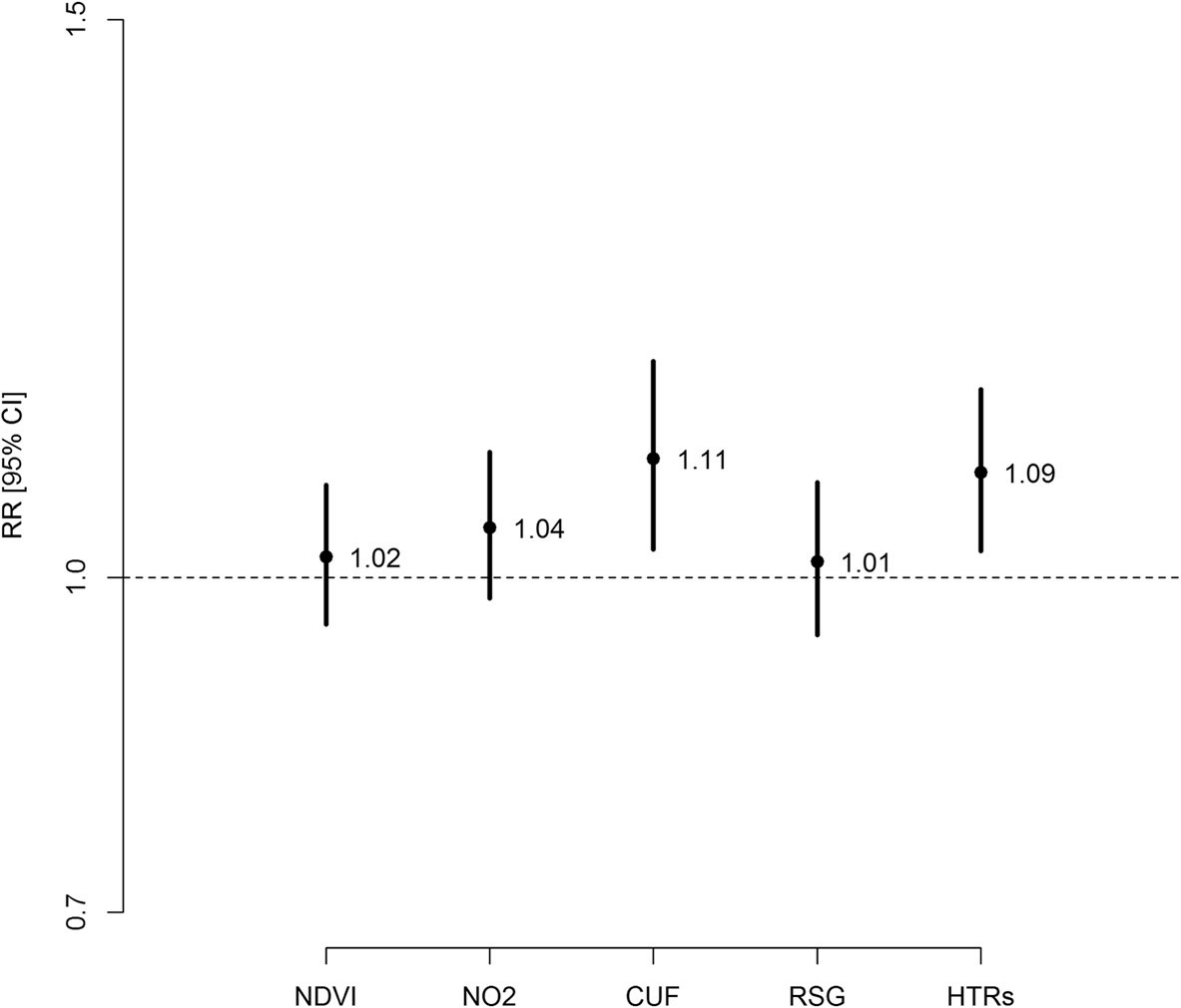
Estimated rate ratios (RR) and 95% confidence intervals from Poisson ridge regression model on symptom score

Estimated rate ratios and 95% CIs from the Poisson ridge regression model for the symptom score are reported in Fig. 3. Children living in proximity to HTRs (≤200 m) had a higher symptom score (RR = 1.09, 95% CI [1.02–1.17]) than children living > 200 m from HTRs. Children living in CUF areas had a higher symptoms score (RR = 1.11, 95% CI [1.03–1.19]) than children living in DUF areas.

The Poisson ridge regression model performed using the full quartile for NDVI and NO_2_ is shown in Additional file 1: Table S3.

## Discussion

Our study is one of the few assessing the association between indicators of urban-related environmental exposures, namely “green”, “grey” and outdoor pollution, with respiratory/allergic and general symptoms in schoolchildren. A very low exposure to greenness measured by NDVI was associated with a higher risk of nasal symptoms. Children living in continuous urban fabric areas had more ocular and general symptoms than those living in discontinuous urban fabric areas. Living in proximity to HTRs was associated with an increased risk of ocular and nasal symptoms. Extreme exposure to NO_2_ ≥ 60 μg/m^3^ (4th quartile) was associated with a higher risk of general symptoms.

The association between urban green spaces and health effects in children has been recently addressed, but inconsistent findings have been found, particularly in relation to respiratory and allergic conditions. Lovasi et al., in a cross-sectional and ecological study involving 4-year-old and 5-year-old children from 42 areas throughout New York City, showed that one standard deviation increase in tree density was associated with a lower asthma prevalence [7]. In a more recent cross-sectional study using different measures for evaluating exposure to greenness, Davdand et al. found no association between current asthma and residential surrounding greenness when measured through NDVI and proximity to a forest, whereas a 60% higher prevalence of current asthma was found to be associated with living close to a park. In the same study, current allergic rhino-conjunctivitis was not associated with NDVI, but was positively associated with residential proximity to a park or to a forest, although none of the associations was statistically significant [12].

Differently from other studies, we found that very low (1st quartile) exposure to greenness measured by NDVI, an objective measure capturing even small-scale green spaces, was associated with a higher risk of self-reported nasal symptoms. We hypothesized that this finding might be ascribed to the lack of mitigation effect of greenness on pollutants from vehicular sources, such as diesel exhaust particles (DEP) recently shown to be involved in the oxidative stress-mediated pathway leading to the dysfunction of epithelial barriers of nasal mucosa [41]. However, we do underline that these results have to be considered with caution and cannot be generalized since NDVI is not able to identify which, if any, particular vegetation types, pathways of exposure or duration of exposures are present [9].

Living in urban areas seems a driving factor for the increase of non-communicable diseases, with the highest burden on children. Only few studies have assessed the effect of greyness on respiratory problems in children, showing that a higher exposure to residential surrounding greyness was associated with an increased risk of bronchitis in the Mediterranean area [19]. Similarly, Ebisu et al. found that increased urban land use near a family’s residence was associated with severity of respiratory symptoms like wheezing among infants [42]. On the contrary, no statistically significant associations between greyness and bronchitis, wheezing, asthma and allergic rhinitis was found in the pooled data of two European birth cohorts of children up to 4 years of age from the Euro-Siberian and Mediterranean regions in Spain [18].

In the present study, we chose the term “greyness” in order to globally include variables such as industrial areas, dumps and construction sites, which define the built environment at a general (CORINE) as well as at an individual (RSG) level [30]. The application of CLC, which is suitable for analyzing land use dynamics at the national or regional level, allowed us to establish an association between greyness and self-reported ocular and general symptoms in children even at small-scale. This result suggests that CLC might be used as an environmental health determinant in research settings.

Traffic–related air pollution has been consistently associated with the occurrence and/or exacerbation of respiratory symptoms in children. We found an increased risk of self-reported ocular and nasal symptoms in children living in proximity to HTRs, in accordance with data from a cross-sectional study by Porebski et al., reporting increased frequency of current nasal and ocular symptoms in adolescents residing within 200 m of a major roadway [43].

Although our results might be affected by the self-reported answers from the questionnaire, it is to be pointed out that an increased time-trend prevalence in metropolitan areas has already been described in the SIDRIA studies conducted in Italy, at least for rhino-conjunctivitis [24]. As expected, based on the chosen study site (within the background monitoring station area), we did not find significant associations between self-reported pulmonary symptoms and living in proximity to HTRs. This finding is in line with the results of a cross-sectional study published by Rosenlund et al. where, in Italian children aged 9–14 years, none of the self-reported respiratory symptoms (asthma, wheezing, cough, phlegm and rhinitis) was positively associated with distance from busy roads [26].

Similarly to Rosenlund’s study in Central Italy, we did not find any association between NO_2_ levels and pulmonary symptoms in our children residing in Southern Italy, emphasizing that outdoor NO_2_ effects on pulmonary symptoms remain controversial in population-based children studies [26, 44, 45]. However, some authors suggest that the observed effects are possibly due to co-pollutants, other than NO_2_ alone, such as sulfur dioxide and ozone [46].

Associations between some of the studied environmental risk factors persisted even when considering the symptom score. In particular, the latter significantly increased in children living in proximity to HTRs and in those living in CUF. These results are in agreement with numerous studies demonstrating that elevated concentrations of traffic-related air pollutants in the near-road environment are associated with numerous adverse human health effects [47–49].

The main strength of our study is the simultaneous use of multiple indices for the estimation of greenness, greyness and outdoor pollution exposure. Another strength is that information on symptoms was provided through well-validated questionnaire-based definitions. Furthermore, the use of the LUR model can be considered as a strength since it has been extensively used in previous studies, showing reliable estimates [45, 50]. Lastly, we adjusted for known and potential confounders/effect modifiers, including gender, age, FSES, atopy, doctor diagnosed asthma, parental history of allergy and preterm birth to account for residual confounding.

Our study also has some limitations. Firstly, we did not perform any objective evaluation of lung conditions, such as respiratory function and measures of oxidative stress-induced inflammation, which has been suggested as a relevant mechanism underlying the respiratory health effects of air. Secondly, we did not evaluate some risk factors specifically associated with urbanization, such as noise and stress, which may negatively affect children’s health. Another limitation might be related to the small sample size and to the fact that all the children lived within the same city area; nonetheless, some relevant associations with environmental risk factors were found. Lastly, the cross-sectional design of the current study could be considered a limitation; a longitudinal study may make it possible to confirm the present findings.

## Conclusions

In the current study, a comprehensive assessment of urban-related environmental exposures on respiratory/allergic and general symptoms in schoolchildren was performed using multiple indices for the estimation of greenness, greyness and outdoor pollution. Multiple exposures related to greenness, greyness (measured by CORINE) and air pollution within the urban environment are associated with respiratory/allergic and general symptoms in schoolchildren. Conversely, no association was found when considering the individual exposure to greyness measured using the RSG indicator,

Indeed, the observed association between greyness (measured by CORINE) and children’s health emphasizes the need of recommending certain exposures to be refined as well as of sensitizing stakeholders to elaborate sustainable and child-friendly urban planning.

Even though the role of greenness in reducing air pollution is controversial, theaforementioned association supports promotion and implementation of nature-based solutions (e.g. green roofs, to increase urban green by transforming flat roofs of existing buildings into green areas; near-road vegetation barriers) as a potential mitigation strategy for air pollution [51, 52] .

Further research is warranted in order to disentangle the complex relationships between different coexisting factors within the urban environment which may affect children’s health. An improved awareness of urban-related risks on health might help in implementing policies within the novel context of “urban health”, aimed at supporting sustainable development for most children who will live in the cities of the future.

## Additional file

Additional file 1: **Table S1.** Risk factors for self-reported ocular, nasal, pulmonary and general symptoms from children questionnaires: estimated odds ratios (OR) and 95% confidence intervals (95% CI) from univariable logistic regression models. **Table S2.** Risk factors for self-reported ocular, nasal, pulmonary and general symptoms from children questionnaires: estimated odds ratios (OR) and 95% confidence intervals (95% CI) from multivariable logistic ridge regression models using the full quartile for NDVI and NO_2._ **Table S3** Multivariable Poisson ridge regression models, using the full quartile for NDVI and NO2, for symptoms score: estimated rate ratios (RR) and 95% confidence intervals (95% CI). (DOCX 25 kb)

## Abbreviation

CPSE: Current Passive Smoke Exposure
CUF: Continuous urban fabric areas
DUF: Discontinuous Urban Fabric
FSES: Family’s Socio-Economic Status
HTRs: High traffic roads
NDVI: Normalized Difference Vegetation Index
NO2: Nitrogen dioxide
RSG: Residential Surrounding Greyness
