# Supplementary material for: Associations of greenness, greyness and air pollution exposure with children’s health: a cross-sectional study in Southern Italy
Source: Environ Health. 2018 Dec 5;17:86. doi: 10.1186/s12940-018-0430-x (PMC6282291; doi:10.1186/s12940-018-0430-x)
Supplement: Supplementary file 1 — Table S1. Risk factors for self-reported ocular, nasal, pulmonary and general symptoms from children questionnaires: estimated odds ratios (OR) and 95% confidence intervals (95% CI) from univariable logistic regression models. Table S2. Risk factors for self-reported ocular, nasal, pulmonary and general symptoms from children questionnaires: estimated odds ratios (OR) and 95% confidence intervals (95% CI) from multivariable logistic ridge regression models using the full quartile for NDVI and NO2. Table S3 Multivariable Poisson ridge regression models, using the full quartile for NDVI and NO2, for symptoms score: estimated rate ratios (RR) and 95% confidence intervals (95% CI). (DOCX 25 kb) [file 12940_2018_430_MOESM1_ESM.docx]

**Supplementary material**

Table S1. Risk factors for self-reported ocular, nasal, pulmonary and general symptoms from children questionnaires: estimated odds ratios (OR) and 95% confidence intervals (95% CI) from univariable logistic regression models.

|  |  |  |  |  |  |  |
| --- | --- | --- | --- | --- | --- | --- |
|  | Ocular symptoms | Nasal symptoms | | Pulmonary symptoms | | General symptoms |
|  | OR [95% CI] | | OR [95% CI] | | OR [95% CI] | OR [95% CI] |
| *NDVI >0.15 (reference)* | *1.00* | *1.00* | | *1.00* | | *1.00* |
| NDVI ≤0.15 (1^st^ quartile) | 1.40 [0.77-2.53] | **2.23 [1.05-4.76]** | | 0.90 [0.45-1.80] | | 1.01 [0.40-2.59] |
| *DUF (reference)* | *1.00* | *1.00* | | *1.00* | | *1.00* |
| CUF | **2.94 [1.33-6.55]** | 1.49 [0.64-3.48] | | 0.91 [0.40-2.05] | | 3.07 [0.78-12.1] |
| *No RSG (reference)* | *1.00* | *1.00* | | *1.00* | | *1.00* |
| RSG (300-m buffer) | 0.86 [0.50-1.47] | 1.13 [0.61-2.07] | | 0.95 [0.51-1.76] | | 1.17 [0.49-2.75] |
| *HTRs>200m (reference)* | *1.00* | *1.00* | | *1.00* | | *1.00* |
| HTRs≤200m | **3.07 [1.80-5.28]** | **2.16 [1.17-3.96]** | | 0.73 [0.40-1.35] | | 1.65 [0.70-3.88] |
| *NO_2_ <60 µg/m^3^ (reference)* | *1.00* | *1.00* | | *1.00* | | *1.00* |
| NO_2_ ≥60 µg/m^3^ (4^th^ quartile) | 1.25 [0.68-2.27] | 1.29 [0.64-2.60] | | 1.13 [0.57-2.23] | | **5.01 [1.33-18.9]** |

Definition of abbreviations: NDVI: Normalized Difference Vegetation Index; DUF: Discontinuous Urban Fabric; CUF: Continuous Urban Fabric; RSG: Residential Surrounding Greyness; HTRs: High traffic roads; NO_2_: Nitrogen dioxide. Significant effects are in bold. Values represent odds ratios, with 95% confidence intervals shown in parentheses.

Table S2. Risk factors for self-reported ocular, nasal, pulmonary and general symptoms from children questionnaires: estimated odds ratios (OR) and 95% confidence intervals (95% CI) from multivariable logistic ridge regression models using the full quartile for NDVI and NO_2_

|  | Ocular Symptoms | | Nasal Symptoms | | Pulmonary Symptoms | | General Symptoms | |  |
| --- | --- | --- | --- | --- | --- | --- | --- | --- | --- |
|  | OR* | 95% CI | OR* | 95% CI | OR* | 95% CI | OR* | 95% CI |  |
| **NDVI** |  |  |  |  |  |  |  |  |  |
| 2^nd^ quartile vs 1^st^ quartile | **0.78** | **[0.61-1.00]** | 0.89 | [0.74-1.06] | **0.80** | **[0.66-0.98]** | 1.05 | [0.78-1.41] |  |
| 3^rd^ quartile vs 1^st^ quartile | 0.89 | [0.69-1.14] | 0.89 | [0.74-1.07] | 1.29 | **[**0.98**-**1.56**]** | 0.75 | [0.54-1.03] |  |
| 4^th^ quartile vs 1^st^ quartile | 1.17 | [0.92-1.50] | 0.99 | [0.84-1.17] | 0.97 | [0.78-1.20] | 0.78 | [0.61-1.01] |  |
| CUF | **1.45** | **[1.10-1.91]** | 1.08 | [0.87-1.34] | 0.98 | [0.76-1.26] | 1.30 | **[**0.98-1.70**]** |  |
| RSG | 0.98 | [0.80-1.21] | 1.06 | [0.91-1.23] | 0.99 | [0.81-1.20] | 1.07 | [0.80-1.46] |  |
| HTRs≤200m | **1.59** | **[1.27-1.98]** | **1.22** | **[1.05-1.42]** | 0.92 | [0.76-1.11] | 1.16 | [0.86-1.56] |  |
| **NO_2_** |  |  |  |  |  |  |  |  |  |
| 2^st^ quartile vs 1^st^ quartile | 0.88 | [0.70-1.13] | 0.95 | [0.80-1.13] | 0.98 | [0.81-1.20] | 0.89 | [0.62-1.26] |  |
| 3^rd^ quartile vs 1^st^ quartile | 0.99 | [0.78-1.25] | 0.98 | [0.83-1.17] | 0.93 | [0.76-1.13] | 0.77 | [0.54-1.11] |  |
| 4^th^ quartile vs 1^st^ quartile | 1.14 | [0.91-1.44] | 1.06 | [0.90-1.25] | 1.02 | [0.83-1.25] | **1.45** | **[1.18-1.78]** |  |

Definition of abbreviations: NDVI: Normalized Difference Vegetation Index; DUF: Discontinuous Urban Fabric; CUF: Continuous Urban Fabric; RSG: Residential Surrounding Greyness; HTRs: High traffic roads; NO_2_: Nitrogen dioxide. Significant effects are in bold. Values represent odds ratios, with 95% confidence intervals shown in parentheses. *Accounting for gender, age, FSES, atopy, doctor diagnosed asthma, parental history of allergy and preterm born. Values of quartile are for NDVI: 1^st^=[0,0.15), 2^nd^[0.15,0.234), 3^rd^=[0.234,0.351), 4^th^= [0.351,0.78), for NO2 1^st^=[0, 31.99), 2^nd^[31.99, 49.97), 3^rd^=[49.97,60.21), 4^th^= [60.21, 105.63)

Table S3. Multivariable Poisson ridge regression models, using the full quartile for NDVI and NO2, for symptoms score: estimated rate ratios (RR) and 95% confidence intervals (95% CI)

|  | Symptom score | |  |
| --- | --- | --- | --- |
|  | RR* | 95% CI |  |
| **NDVI** |  |  |  |
| 2^nd^ quartile vs 1^st^ quartile | 0.95 | [0.88-1.01] |  |
| 3^rd^ quartile vs 1^st^ quartile | 0.99 | [0.92-1.06] |  |
| 4^th^ quartile vs 1^st^ quartile | 1.04 | [0.98-1.10] |  |
| CUF | **1.10** | **[1.03-1.18]** |  |
| RSG | 1.01 | [0.96-1.07] |  |
| HTRs≤200m | **1.08** | **[1.02-1.15]** |  |
| **NO_2_** |  |  |  |
| 2^st^ quartile vs 1^st^ quartile | 1.00 | [0.95-1.06] |  |
| 3^rd^ quartile vs 1^st^ quartile | 0.96 | [0.90-1.02] |  |
| 4^th^ quartile vs 1^st^ quartile | 1.04 | [0.98-1.11] |  |

Definition of abbreviations: NDVI: Normalized Difference Vegetation Index; DUF: Discontinuous Urban Fabric; CUF: Continuous Urban Fabric; RSG: Residential Surrounding Greyness; HTRs: High traffic roads; NO_2_: Nitrogen dioxide. Significant effects are in bold. Values represent rate ratios, with 95% confidence intervals shown in parentheses. *Accounting for gender, age, FSES, atopy, doctor diagnosed asthma, parental history of allergy and preterm born. Values of quartile are for NDVI: 1^st^=[0,0.15), 2^nd^[0.15,0.234), 3^rd^=[0.234,0.351), 4^th^= [0.351,0.78), for NO2 1^st^=[0, 31.99), 2^nd^[31.99, 49.97), 3^rd^=[49.97,60.21), 4^th^= [60.21, 105.63)
